# Carbamazepine-induced acute generalized exanthematous pustulosis triggering diabetic ketoacidosis in a patient with type II diabetes mellitus: a case report

**DOI:** 10.1097/MS9.0000000000004623

**Published:** 2025-12-23

**Authors:** Bijay Bastola, Lok Raj Bohora

**Affiliations:** a Pokhara Academy of Health Sciences, Pokhara, Nepal; b Mahakali Provincial Hospital, Nepal

**Keywords:** acute generalized exanthematous pustulosis, adverse drug reaction, carbamazepine, diabetic ketoacidosis, multidisciplinary management

## Abstract

**Introduction and Importance:**

Acute generalized exanthematous pustulosis (AGEP) is a rare, severe cutaneous adverse drug reaction, typically triggered by antibiotics or anticonvulsants. Although carbamazepine is an uncommon cause, it was identified as the culprit in this case. Uniquely, the AGEP episode was complicated by diabetic ketoacidosis (DKA) in a patient with uncontrolled type II diabetes mellitus, with no identifiable trigger apart from the severe systemic inflammation associated with AGEP.

**Case Presentation:**

A 54-year-old patient with multiple comorbidities developed AGEP shortly after initiating carbamazepine therapy for poststroke seizure. The condition was complicated by the onset of DKA. Systemic corticosteroids were administered to control cutaneous symptoms but caused transient worsening of glycemic control.

**Case Discussion:**

Discontinuation of carbamazepine combined with standard DKA treatment resulted in full recovery. The case highlights the complexity of managing AGEP triggered by carbamazepine, especially when complicated by metabolic disturbances like DKA.

**Conclusion:**

Carbamazepine-induced AGEP is an uncommon but serious reaction, which, in rare cases, can lead to DKA. The presence of both conditions simultaneously complicates treatment, as interventions for one may worsen the other. Effective management of these overlapping issues, particularly in patients with multiple health concerns, requires a cautious and collaborative approach.

## Introduction

Acute generalized exanthematous pustulosis (AGEP) is a rare, drug-induced skin reaction characterized by the abrupt onset of numerous small, sterile pustules on an erythematous base. It is classified as a severe cutaneous adverse reaction (SCAR) with an estimated incidence of one to five cases per million people annually and a mortality rate of approximately 1–2%^[1,2]^.

Carbamazepine, although widely used as an anticonvulsant, is an uncommon trigger for AGEP. It is more frequently associated with anticonvulsant hypersensitivity syndrome (AHS), a potentially fatal reaction marked by fever, rash, lymphadenopathy, hepatitis, leukocytosis, and eosinophilia. AGEP is a rare presentation within the spectrum of AHS^[3]^.

**HIGHLIGHTS**

- Carbamazepine-induced acute generalized exanthematous pustulosis (AGEP) is a rare, severe cutaneous drug reaction.
- AGEP unusually precipitated diabetic ketoacidosis in this patient.
- The coexistence of AGEP and DKA created a complex therapeutic challenge.
- Systemic steroids helped control AGEP, but complicated glucose management.
- Multidisciplinary care was crucial for successful treatment and recovery.

It is commonly seen following the introduction of the drug and has a certain genetic predisposition, with studies identifying increased frequencies of HLA-B51, HLA-DR11, and HLA-DQ3 in affected individuals^[4]^.

We report the case of a 54-year-old male who developed AGEP following carbamazepine initiation. Uniquely, this case was also complicated by diabetic ketoacidosis (DKA) in a patient with type II diabetes mellitus, without other apparent triggers. To our knowledge, this may represent the first reported instance of DKA precipitated by a drug-induced skin reaction, underscoring the importance of early recognition and multidisciplinary management in patients with complex comorbidities. This case report is written according to the CARE guideline and TITAN reporting standards to ensure a standard and comprehensive presentation of the case^[5,6]^.

## Case description

**Figure 1.**
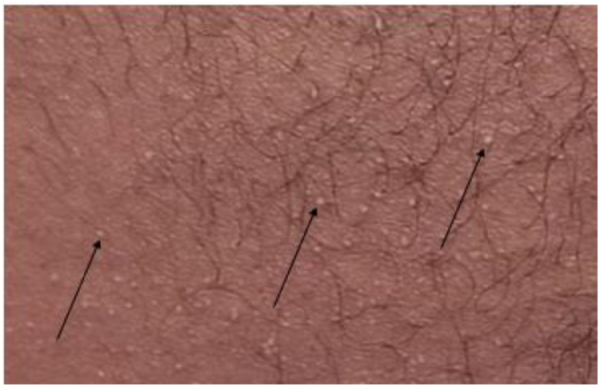
Multiple white pinpoint non-follicular pustules with an erythematous background.

A 54-year-old male presented to the emergency department of our tertiary care hospital with complaints of an acute and progressively worsening generalized pruritic rash, accompanied by shortness of breath. The eruption appeared on the abdomen and axillary region and rapidly progressed over 2 days to involve the limbs and face. The patient reported that the rash began as discrete lesions, which later became confluent, with the subsequent development of widespread yellowish-white pinpoint dots over the affected areas (Fig. 1).

In addition to the rash, the patient experienced intermittent fever that began around the same time as the skin lesions. The initial febrile episode lasted for approximately 4 days and resolved spontaneously, but recurred 2 days prior to hospital admission with similar intermittent characteristics.

On the day of admission, he developed acute-onset dyspnea at rest. It was not associated with chest pain, sweating, calf swelling, dizziness, or syncope. Furthermore, he complained of nonspecific generalized abdominal discomfort.

His past medical history was significant for type II diabetes mellitus for 12 years, hypertension for 9 years, hypothyroidism for 6 years, a hemorrhagic cerebrovascular accident (left thalamic bleed) 6 months prior, and a poststroke seizure disorder. Blood pressure and thyroid function were well controlled; however, glycemic control remained suboptimal despite multiple medications, as reflected by an HbA1c of 9.3%. His ongoing medications included levo-thyroxine, dapagliflozin, metformin, glimepiride, rosuvastatin, amlodipine-telmisartan, and carbamazepine. Notably, carbamazepine was initiated 15 days prior to hospital presentation for the management of poststroke seizures.

On initial evaluation, the patient’s vital signs were notable for tachycardia, with a heart rate of 106 beats per minute, and tachypnea, with a respiratory rate of 24 breaths per minute, and fever with axillary temperature of 100.2°F, while blood pressure was within normal limits. The glucometer revealed a reading as high (>500 mg/dl).

**Figure 2.**
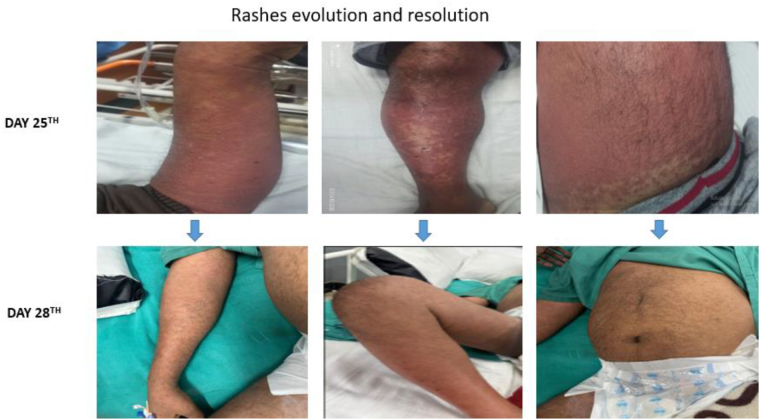
Widespread erythematous pustular rash over trunk and limbs, with resolution beginning on day 3 after carbamazepine withdrawal and supportive treatment.

On systemic examination, the abdomen was soft, non-distended, and diffusely tender, while the respiratory, cardiovascular, and nervous system examinations were normal. Dermatological examination revealed generalized erythematous confluent rashes with non-follicular pustules, without any exfoliations; rashes were non-blanchable, non-painful (Fig. 2). Ear, nose, and throat examination revealed no angioedema; no lesions were seen in the oral mucosa, eyes, and anogenital area. Following a thorough dermatological examination, a preliminary diagnosis of carbamazepine induced AGEP was made. His lab report is tabulated as below: Table 1.

**Table 1:** Lab parameters at the time of admission

Test/parameter	Result	Reference range	Remarks
**CBC**	–	–	–
WBC	**22.84 × 10^3^**	4500–11 000/cu mm	**Leukocytosis**
Neutrophil	95%	40–80%	**Neutrophilia**
Eosinophil	1%	1–6%	Normal
**RBS**	**881**	70–140 mg/dl	**Hyperglycemia**
**RFT**	–	–	–
Sodium (Na)	122 mmol/l	135–145	**Hyponatremia**
Corrected sodium(Na) (katz formula)	**134** mmol/l	135–145	**Hyponatremia**
Potassium (K)	4.78 mmol/l	3–5	Normal
Creatinine (Cr)	**3.0** mg/dl	0.7–1.3	**Increased**
Urea (Ur)	**172** mg/dl	7–20	**Increased**
**ABG**	–	–	–
pH	**7.28**	7.35–7.45	**Low (acidosis)**
pCO₂	30.2 mmHg	35–45	Slightly low (respiratory compensation)
pO₂	89.8 mmHg	75–100	Normal
Lactate	2.8 mmol/l	0.5–1.5	Mildly elevated
HCO₃^−^	**14.13** mEq/l	22–26	**Low (metabolic acidosis**)
Anion Gap	**22.25**mEq/l	8–16	**High anion gap**
**LFT**	–	–	–
Total protein	5.4 gm/dl	6.3–8.2 g/dl	Decreased
Albumin	3.3 gm/dl	3.5–5.0 gm/dl	Decreased (negative acute phase reactant)
Total bilirubin	1.57 mg/dl	1.2–2.5 mg/dl	Normal
Conjugated bilirubin	0.2 mg/dl	0.0–0.3 mg/dl	Normal
AST	20 U/l	17–59 U/l	Normal
ALT	47 U/l	0–50 U/l	Normal
**Urine R/M/E**	–	–	–
Urine ketone	**1+**	Nil	**Ketonuria**
Sugar	**4+**	Nil	**Glycosuria**
Pus cells	1–2 cells/hpf	1–3 cells/hpf	Normal
Protein	**Trace**	Nil	**Trace proteinuria**
Hba1c	**9.3%**	<5.7%	**Increased**

In the background of a high glucose level measuring 881 mg/dl, low bicarbonate 14.13 mmol/l, low pH of 7.28, and positive urine ketone, a diagnosis of DKA was made.

Blood culture was negative for any growth. The swab sent from the pustules reveals growth of normal skin flora. The urine routine examination revealed significant glucosuria, with sugar measured at 4+, while only a trace amount of protein was detected. Microscopy showed one to two pus cells per high-power field and no red blood cells. Imaging studies, including a chest X-ray and ultrasonography of the abdomen and pelvis, demonstrated no abnormalities, with all findings reported as within normal limits. Due to concerns regarding the patient’s unstable condition, the patient’s family declined to provide consent for the skin biopsy. In the background of generalized inflammatory eruptions with no apparent source of infection, a diagnosis of carbamazepine induced AGEP with DKA was made.

Given the clinical scenario, several differential diagnoses were considered, including DRESS, SJS/TEN, generalized pustular psoriasis, and sepsis-induced pustulosis. Although the patient developed a widespread rash about 13 days after starting the drug and had acute kidney injury, DRESS was initially suspected. However, retrospective assessment of the RegiSCAR score was low (1), and the patient improved quickly after stopping the drug – all findings arguing against DRESS. SJS/TEN was similarly ruled out due to the absence of mucosal involvement, absence of bullae, lack of targetoid lesions, no evidence of epidermal detachment, and a negative Nikolsky sign – features inconsistent with SJS/TEN. Generalized pustular psoriasis was excluded based on its typically chronic or recurrent course, the common presence of personal or family history of psoriasis (none evident in this case), and the characteristic “lakes of pus,” which were not observed. Additionally, the rash resolved completely within 15 days following discontinuation of the offending drug, whereas in generalized pustular psoriasis, resolution generally occurs over weeks to months, further supporting a diagnosis other than psoriasis. Sepsis-induced pustulosis was also considered unlikely, as no infectious source was identified and the onset of symptoms closely followed the introduction of a new drug rather than an infection.

The patient was admitted to the ICU for close monitoring and further management. Considering the high mortality rate in DKA patients, the case was managed as per standard protocol with IV fluids and insulin infusion. As an initial measure, carbamazepine was stopped, and valproate and lorazepam were used as substitutes for carbamazepine for seizure control, following consultation with neuropsychiatry.

For AGEP, injection of dexamethasone 8 mg IV stat was given in the emergency room, followed by 4 mg IV twice daily. It was continued for 3 days. Due to the coadministration of dexamethasone, the maintenance of blood sugar became challenging; hence, hourly GRBS monitoring was done. However, due to rapid fluctuation of blood glucose levels, it was stopped, and the patient was continued on topical steroid cream. In addition to it, an antihistamine medication was given twice daily.

On the third day of admission, following endocrine consultation, insulin infusion and IV fluid were stopped, and an oral diet was allowed to the patient. The insulin regimen was changed to a basal-bolus regimen with insulin glargine and regular insulin for the rest of the hospital stay.

Follow-up investigations, including arterial blood gas (ABG), complete blood count (CBC), and renal function tests (RFT), demonstrated resolution of metabolic acidosis, normalization of renal parameters, and a decrease in leukocyte count. These findings reflected clinical improvement in DKA, prerenal acute kidney injury (AKI), and systemic inflammation secondary to drug-induced hypersensitivity, following adequate fluid resuscitation, prompt withdrawal of the offending agent, and appropriate supportive management.

**Figure 3.**
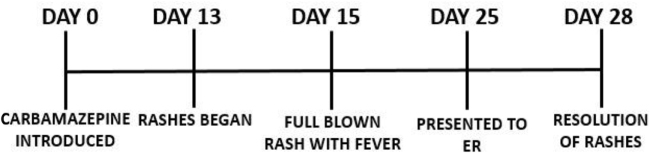
Disease progression timeline.

The patient demonstrated progressive improvement in cutaneous lesions, with reduction in erythema, diminution of pustules, and onset of scaling observed from the third day of admission. Complete resolution of the rash was noted by the fifth day (Fig. 3). A EuroSCAR AGEP score of 9, calculated on the fifth day of admission, confirmed the diagnosis of carbamazepine-induced AGEP (Table 2).

**Table 2:** Euro SCAR AGEP scoring system^[7]^

Feature	Score
Fever (≥38°C)	+1
Resolution within 15 days	0
Histology (spongiform pustules)	–
Neutrophils (≥7000/mm^3^)	+3
Pustules(typical)	+2
Erythema(typical)	+2
Distribution	+2
Postpustular desquamation	+1
Mucosal involvement	0
Acute onset (≤10 days)	−2

Total score – 9 (definite diagnosis of AGEP).

During the follow-up visit, the patient was advised to undergo a patch test to confirm hypersensitivity to carbamazepine. However, he declined the test, citing concerns about the potential for severe drug reactions and the need for referral to a specialized center.

## Discussion

AGEP is a rare but well-characterized cutaneous adverse reaction, typically drug-induced. Its incidence is estimated at approximately one to five cases per million individuals each year^[8]^. While AGEP can affect people across all ages, a slight female predominance has been documented in several studies^[9]^.

Roughly 90% of AGEP cases are attributed to medications^[10]^. Common culprits include beta-lactam and macrolide antibiotics, as well as drugs such as hydroxychloroquine, antivirals, antifungals, antiparasitics, chemotherapy agents, antirheumatics, analgesics, anticonvulsants, and intravenous contrast media. Although carbamazepine is infrequently cited as a trigger, few cases have been reported in the literature where AGEP has been triggered by carbamazepine^[11–13]^.

Clinically, AGEP is characterized by a sudden onset of numerous small, noninfectious pustules on a background of erythema, often accompanied by itching, burning sensations, and fever. The eruption typically starts in skin folds and spreads rapidly to involve the trunk, extremities, and face, usually sparing the palms and soles. Mucosal involvement is uncommon and generally limited to the oral mucosa. The pustules tend to desquamate over 1–2 weeks, with resolution typically occurring within 15 days following discontinuation of the offending agent^[7]^.

In this particular case, the patient developed an acute pustular eruption shortly after beginning treatment with carbamazepine. The presentation included fever, neutrophilia, and widespread pustules, without evidence of infection, prompting early suspicion for AGEP. Diagnosis was based on clinical features and confirmed using the EuroSCAR criteria, which assess various parameters to determine the likelihood of AGEP^[7]^. The findings met the threshold for a definitive diagnosis. However, considering that carbamazepine, an antiepileptic drug, is a common trigger for DRESS than AGEP with onset of erythematosus rashes over a period of 2 weeks, with an organ involvement in the form of acute renal injury, a possible overlap syndrome was considered during the time of presentation. However, A RegiSCAR score calculated retrospectively, with a value of only 1, was consistent with not a case of DRESS.

Our case demonstrates notable similarities with previously reported instances of carbamazepine-induced AGEP. Goh et al. described a 28-year-old Chinese woman who developed AGEP with SJS/TEN overlap 13 days after initiating carbamazepine, requiring corticosteroids and intravenous immunoglobulin. Similarly, Son et al. reported a 41-year-old man who developed AGEP approximately 1 month after starting carbamazepine, managed with oral prednisolone. Although the onset of AGEP typically occurs within the first 10 days of exposure to the inciting drug, delayed presentations, as observed in these cases and in our patient, suggest a slightly atypical temporal pattern in carbamazepine-induced AGEP.

**Figure 4.**
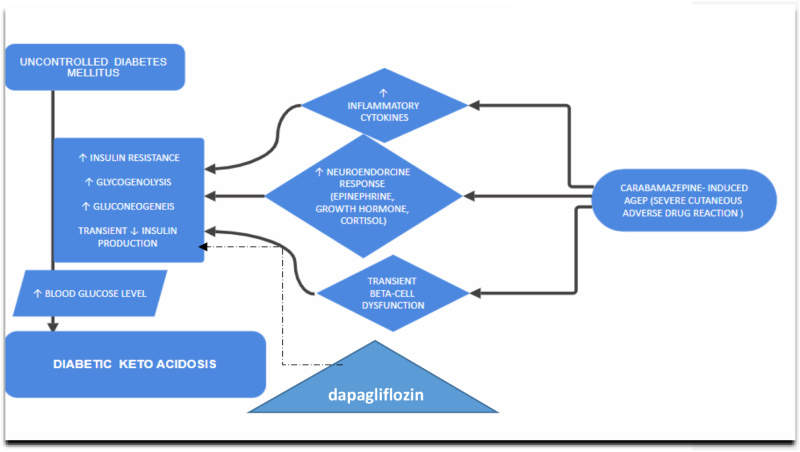
Proposed mechanism of DKA triggered by carbamazepine-induced AGEP with dapagliflozin as a permissive factor in the above-mentioned case of uncontrolled diabetes mellitus.

What made this case particularly unusual was the simultaneous onset of DKA in a patient with type II diabetes mellitus, without any other clear precipitating factors. Although the patient was on dapagliflozin, an SGLT2 inhibitor often associated with euglycemic DKA, the pattern is not typical of SGLT2 inhibitor-induced ketoacidosis. The abrupt rise in blood glucose from an average of ~220.21 mg/dl (corresponding HbA1c of 9.3%) to 881 mg/dl, occurring in parallel with a SCAR, strongly suggests that a drug reaction was a precipitating factor. While SGLT2 inhibitors lower glucose and insulin and modestly raise glucagon, these effects are unlikely to produce such extreme hyperglycemia. At most, dapagliflozin may have had a permissive rather than primary role in promoting ketosis. In the background of SCAR, the presumed mechanism involves an inflammatory cascade, including elevated cytokines and stress hormones, along with transient β-cell dysfunction, culminating in severe hyperglycemia and ketoacidosis^[14,15]^ (Fig. 4).

A PubMed and Google Scholar search using the MeSH terms “Pustulosis, Acute Generalized Exanthematous” AND “Diabetic Ketoacidosis” AND “Drug Eruptions” did not identify any prior reports, highlighting the uniqueness of this case.

Initial treatment focused on standard DKA protocols, intravenous insulin, fluid replacement, and close glucose monitoring. However, a concern for possible overlap with DRESS prompted the use of systemic steroids. Systemic corticosteroids in DRESS are indicated in patients with severe organ involvement (any one of liver, kidney, lung, heart), life-threatening complications (e.g., hemophagocytosis, encephalitis, respiratory failure), or severe disease with confirmed viral reactivation, often in combination with IVIG (Intravenous immunoglobulin) and/or antivirals depending on severity^[16]^. For this reason, systemic corticosteroid therapy was initiated.

Systemic corticosteroids caused significant hyperglycemic fluctuations, increasing insulin requirements and complicating DKA management, which relies primarily on fluid resuscitation and suppression with insulin. Careful risk–benefit assessment allowed rapid tapering of systemic steroids once systemic and cutaneous symptoms stabilized, with high-potency topical therapy maintaining skin control while minimizing further disruption of glycemic stability. Following cessation of carbamazepine and initiation of supportive therapy, the skin eruption completely resolved within 15 days, consistent with the typical AGEP timeline. DKA was successfully treated, and no subsequent metabolic events occurred during the hospital stay.

This case emphasizes the importance of promptly identifying AGEP, especially when caused by less common agents like carbamazepine. Although most AGEP cases can be managed conservatively with drug withdrawal, topical steroids, antipyretics, and antihistamines, systemic corticosteroids may be warranted in severe or complex presentations^[17]^. Their use, however, must be carefully considered due to potential adverse effects, including rare cases of steroid-induced AGEP or paradoxical worsening^[18,19]^. For cases refractory to steroids, alternative immunomodulatory therapies, including biologics, can be used^[17]^.

## Conclusion

In summary, carbamazepine-induced AGEP is a rare and severe adverse drug reaction. The intense inflammatory response triggered by this reaction can, albeit unusually, precipitate DKA. The coexistence of AGEP and DKA presents a therapeutic dilemma, as treatments effective for one condition may exacerbate the other. Managing these overlapping pathologies in a patient with multiple comorbidities is highly challenging. This case underscores the importance of a vigilant, multidisciplinary approach when rare drug reactions intersect with complex systemic illnesses.

## Data Availability

The raw data supporting the conclusions of this article will be made available by the authors without undue reservation.
